# Proton pump inhibitors and the risk of hospital acquired clostridioides difficile infection in critically ill patients

**DOI:** 10.3389/fphar.2026.1829764

**Published:** 2026-07-13

**Authors:** Qian Zeng, Chengfu Guan, Jiana Chen, Li Chen

**Affiliations:** Department of Pharmacy, Nanping First Hospital Affiliated to Fujian Medical University, Nanping, China

**Keywords:** adverse reactions, Clostridioides difficile infection, critically ill, intensive care unit, proton pump inhibitors

## Abstract

**Background:**

Proton pump inhibitors (PPIs) are commonly prescribed medications for critically ill patients. However, the existence of a clear association between PPI prescriptions and Clostridioides difficile infection (CDI) remains controversial. This study aims to investigate the relationship between PPI use and CDI, thereby providing additional evidence for the clinical application of PPIs.

**Methods:**

This study extracted data from the eICU and MIMIC IV databases, ultimately enrolling 26,090 patients. Propensity score matching was employed to balance covariates across groups. Clinical outcomes were compared using χ^2^ tests after matching, and specific risk factors were analyzed via multivariate logistic regression, with results presented as odds ratios and 95% confidence intervals.

**Results:**

Compared with the non-PPI group, the PPI group showed a significantly increased risk of CDI (PPI vs. No-PPI: 2.6% vs. 1.6%; OR, 1.70; 95% CI, 1.43–2.02; p < 0.001). Across all subgroups, the risk of CDI was significantly higher in the PPI group than in the non-PPI group. Obesity (BMI ≥ 30), cancer, diabetes, IBD, kidney disease, and use of high-risk antibiotics were significantly associated with increased CDI risk in patients.

**Conclusion:**

Among critically ill patients, PPI recipients exhibited a significantly increased risk of CDI compared to those not using PPIs.

## 1 Introduction

*Clostridioides difficile* (formerly *Clostridium difficile*) infection (CDI) is caused by a Gram-positive, obligate anaerobic, spore-forming *bacillus* (Lawson et al., 2016). *Clostridioides difficile* is widely distributed in the environment and spreads between hosts via the fecal-oral route. The toxins it produces can cause patients to develop a range of clinical symptoms, including diarrhea, pseudomembranous colitis, sepsis, toxic megacolon, intestinal perforation, and even death (Kuehne et al., 2014; van Prehn et al., 2021). *Clostridioides difficile* is the most common pathogen in the United States, accounting for 12.1% of healthcare-associated infections, with the number of patients increasing annually (Magill et al., 2014). From 2000 to 2010, the number of hospitalizations among nonpregnant adults in the United States due to CDI doubled (Lessa et al., 2015). The economic burden imposed by CDI is equally severe. It is estimated that, in the United States alone, the additional healthcare costs associated with CDI in acute care facilities reach $4.8 billion, with total annual healthcare expenditures resulting from the condition amounting to $6.3 billion (Dubberke and Olsen, 2012; Zhang et al., 2016).

Proton pump inhibitors (PPIs) are among the most commonly prescribed medications, particularly prevalent in Europe and the United States, with increasing trends observed in recent years (Targownik et al., 2022; Torres-Bondia et al., 2022). A review of global trends in PPI use reveals that nearly one in four adults use PPIs, two-thirds of users take high doses, 25% of users continue treatment for over 1 year, and 28% continue treatment for over 3 years (Shanika et al., 2023). In the intensive care unit (ICU), most critically ill patients will receive PPIs to prevent stress ulcers (Barletta et al., 2014). Although PPIs are generally considered safe, controversy has arisen in recent years regarding the relationship between PPI use and certain adverse reactions. A systematic review and meta-analysis incorporating 56 studies (40 case-control and 16 cohort studies) involving 356,683 patients demonstrated a significantly increased risk of CDI among PPI users compared with non-users (Trifan et al., 2017). Another meta-analysis examining the dose-response relationship between PPI usage and CDI risk indicated that the risk of CDI may increase with higher PPI treatment doses and longer duration of therapy (Finke et al., 2025). However, a meta-analysis of 7 randomized controlled trials involving 29,880 patients showed that PPIs did not increase the risk of CDI compared with placebo (Floria et al., 2025). The inconsistencies in existing evidence highlight the current lack of clarity regarding the relationship between PPI use and CDI risk, necessitating further investigation. Therefore, this study aims to analyze a large, multi-center dataset to determine whether PPI use in critically ill patients is associated with an increased risk of CDI, thereby providing additional evidence for the clinical use of PPIs in this population.

## 2 Methods

### 2.1 Data source and population

The data for this study were extracted from the eICU collaborative research database (version 2.0) and MIMIC IV database (version 3.1) (Pollard et al., 2019; Johnson et al., 2024). The eICU database is a multi-center, publicly accessible ICU database containing de-identified, high-granularity medical data from 200,859 ICU admissions across 208 centers in the United States from 2014 to 2015 (Pollard et al., 2018). The MIMIC IV database contains data on over 65,000 ICU patients and more than 200,000 emergency department patients admitted to the emergency department and intensive care unit at Beth Israel Deaconess Medical Center in Boston, Massachusetts, USA, from 2008 to 2022 (Johnson et al., 2023). The eICU and MIMIC-IV databases contain vital signs recorded by healthcare providers during patients’ ICU stays, nursing care plan documentation, measures of disease severity, diagnoses, treatments, laboratory results, and other relevant information. The personnel responsible for data extraction and processing for this study have obtained data usage certification (certification number: 11678655). Informed consent was waived due to the data’s de-identified nature.

**FIGURE 1:**
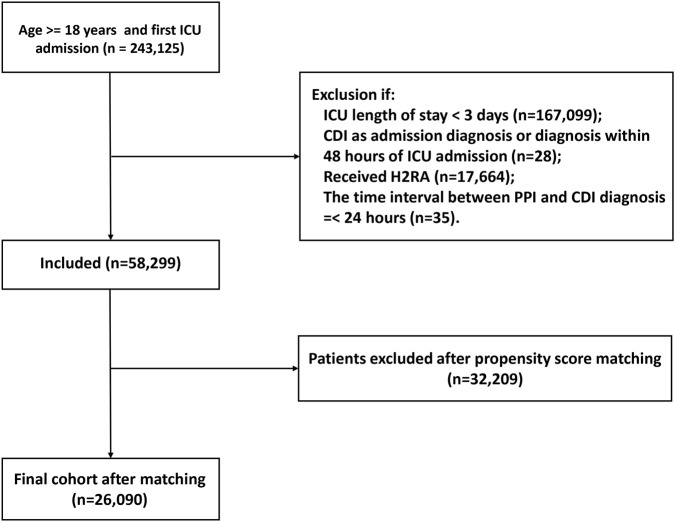
Flowchart of patient selection. Abbreviations: ICU, intensive care unit; CDI, *Clostridioides difficile* infection; H2RA, histamine H2 receptor antagonists; PPI, proton pump inhibitors.

In this study, all patients aged 18 years or older were considered for inclusion. For patients with multiple ICU admissions, only the first admission was considered. Exclusion criteria were as follows: (1) ICU length of stay <3 days; (2) CDI as the admission diagnosis or diagnosis within 48 h of ICU admission; (3) Received H2 receptor antagonists (H2RAs) during the ICU stay; (4) The time interval between PPI and CDI diagnosis ≤ 24 h; (5) Missing data exceeding 30%. Patients who received PPI treatment following the onset of CDI were classified into the non-PPI group. In addition, patients who developed CDI prior to H2RA therapy were retained in the analysis and classified into the non-PPI group. The selection flowchart for the study cohort is shown in Figure 1.

### 2.2 Data extraction

Baseline information collected within 24 h of ICU admission was extracted using Structured Query Language. Demographic information included age, gender, and body mass index (BMI). The patient’s conditions include acquired immunodeficiency syndrome (AIDS), cancer, congestive heart failure (CHF), chronic obstructive pulmonary disease (COPD), dementia, diabetes, inflammatory bowel disease (IBD), and kidney disease. Laboratory indicators include hemoglobin, white blood cell count, albumin, serum creatinine, alanine aminotransferase (ALT), aspartate aminotransferase (AST), and total bilirubin. Treatment administered to patients admitted to the ICU included high-risk antibiotics and immunosuppressants. For variables measured multiple times within 24 h, we selected the maximum value. IBD encompasses ulcerative colitis and Crohn’s disease. High-risk antibiotics refer to cephalosporins, penicillins, clindamycin, fluoroquinolones, and carbapenems. We employ multiple imputation methods to fill in missing values (Zhang, 2016). Multiple imputation generates multiple complete datasets by repeatedly fitting possible values for missing data through a model. Subsequently, it analyzes the generated datasets and combines multiple analytical results to obtain a comprehensive estimate and statistical inference ultimately. Compared to single imputation, multiple imputation fills missing values multiple times, enabling the quantification of uncertainty in missing value estimates and avoiding the illusion of precision (Li et al., 2015). Multiple imputation was performed to generate 20 imputed datasets (M = 20), and the estimates were pooled using Rubin’s rules. Details regarding missing data are provided in Supplementary Figure S1.

### 2.3 Outcomes

The primary outcome of this study was the incidence of new-onset CDI during ICU hospitalization. We use International Classification of Diseases (ICD) codes to identify patients with CDI. The EICU database uses the ICD-9 code (008.45), while the MIMIC database uses both the ICD-9 code (008.45) and the ICD-10 code (A04.7).

### 2.4 Statistical analysis

Continuous variables were tested for normality. Those meeting the criteria for normal distribution were described using mean (standard deviation). For continuous variables not meeting the criteria for normal distribution, median (interquartile range 1–3) was used for description. Categorical variables were described as frequencies (percentages). To compare clinical characteristics between two groups, Student’s t-test was used for continuous variables that met the assumption of normality. At the same time, the Mann-Whitney U test was applied for those who did not. Differences in categorical variables were assessed using the chi-square test or Fisher’s exact test. A two-tailed p-value <0.05 was considered statistically significant.

Using propensity score matching, comparable PPI and non-PPI cohorts were established at a 1:1 ratio to balance baseline confounding factors. A logistic regression model calculated each patient’s propensity score based on baseline characteristics (Table 1) as independent variables and PPI treatment status as the dependent variable. Nearest-neighbor matching was performed on the propensity score table using a 0.05 caliper (Wang et al., 2014). Since propensity-matched datasets are resampled from a population-representative sample—essentially a sample within a sample—hypothesis testing applies to the population from which the sample is drawn. Furthermore, the reduced sample size after propensity matching inherently increases the p-value. Therefore, standardized mean difference (SMD) rather than statistical testing is employed to assess covariate balance within matched cohorts. An SMD ≤0.1 indicates a sufficient balance between the two groups (Austin, 2014).

**TABLE 1:** Baseline characteristics in the two groups before propensity score matching.

Characteristic	No PPI group (n = 45,194)	PPI group (n = 13,105)	P value	SMD
Age	66.0 (55.0–76.0)	66.0 (55.0–76.0)	0.394	0.004
Male, n (%)	24,987 (55.3)	7,383 (56.3)	0.034	0.021
BMI, kg/m^2^	27.7 (23.6–33.3)	27.6 (23.7–32.8)	0.230	0.023
Diagnosed diseases, n (%)				
AIDS	133 (0.3)	59 (0.5)	0.008	0.026
Cancer	3,186 (7.1)	1,694 (12.9)	<0.001	0.197
CHF	7,909 (17.5)	3,558 (27.1)	<0.001	0.232
COPD	5,641 (12.5)	2,998 (22.9)	<0.001	0.275
Dementia	959 (2.1)	418 (3.2)	<0.001	0.066
Diabetes	6,544 (14.5)	3,719 (28.4)	<0.001	0.344
IBD	171 (0.4)	110 (0.8)	<0.001	0.059
Renal disease	4,994 (11.1)	2,467 (18.8)	<0.001	0.219
Laboratory test results				
Hemoglobin, g/dL	11.7 (10.0–13.5)	11.0 (9.5–12.8)	<0.001	0.234
While blood cells, K/uL	12.9 (9.3–18.0)	13.3 (9.4–18.6)	<0.001	0.035
Albumin, g/dL	3.2 (2.7–3.7)	3.1 (2.5–3.6)	<0.001	0.159
Creatinine, mg/dL	1.2 (0.8–1.9)	1.3 (0.9–2.3)	<0.001	0.096
ALT, U/L	26.0 (16.0–50.0)	28.0 (17.0–57.0)	<0.001	0.059
AST, U/L	35.0 (22.0–73.0)	40.0 (24.0–91.0)	<0.001	0.085
Total bilirubin, mg/dL	0.7 (0.4–1.2)	0.8 (0.5–1.5)	<0.001	0.160
Treatments, n (%)				
Metronidazole	5,037 (11.1)	2049 (15.7)	<0.001	0.135
Vancomycin	14,011 (31.0)	4,827 (37.0)	<0.001	0.127
High risk antibiotics	19,652 (43.5)	7,145 (54.5)	<0.001	0.218
Immunosuppression	9,402 (20.8)	3,252 (24.8)	<0.001	0.096

Abbreviations: PPI, proton pump inhibitors; SMD, standardized mean difference; BMI, body mass index; AIDS, acquired immunodeficiency syndrome; CHF, congestive heart failure; COPD, chronic obstructive pulmonary disease; IBD, inflammatory bowel disease; ALT, alanine aminotransferase; AST, aspartate aminotransferase.

In addition, multivariable logistic regression analysis was performed in the overall cohort to explore factors associated with CDI. This analysis was considered exploratory and hypothesis-generating. McNemar tests were used to compare matched clinical outcomes after propensity score matching, with odds ratios (ORs) and 95% confidence intervals (CIs) reported. Bilateral tests with p-values <0.05 were considered statistically significant. Statistical analyses for this study were conducted using R version 4.4.3.

## 3 Results

### 3.1 Baseline characteristics

A total of 58,299 patients were included in the PPI group (n = 13,105) and the non-PPI group (n = 45,194). Before propensity score matching, baseline differences existed between the PPI and non-PPI groups. Detailed demographic and baseline characteristics are presented in Table 1. After propensity score matching, 13,045 patient pairs were successfully matched, with all baseline characteristic differences balanced (SMD <0.1). Detailed baseline information is shown in Table 2.

**TABLE 2:** Baseline characteristics in the two groups after propensity score matching.

Characteristic	No PPI group (n = 13,045)	PPI group (n = 13,045)	SMD
Age	66.0 (55.0–76.0)	66.0 (55.0–76.0)	0.012
Male, n (%)	7,321 (56.1)	7,347 (56.3)	0.004
BMI, kg/m^2^	27.6 (23.5–33.1)	27.6 (23.7–32.8)	0.012
Diagnosed diseases, n (%)			
AIDS	56 (0.4)	59 (0.5)	0.003
Cancer	1707 (13.1)	1,668 (12.8)	0.009
CHF	3,664 (28.1)	3,524 (27.0)	0.024
COPD	3,057 (23.4)	2,961 (22.7)	0.017
Dementia	445 (3.4)	414 (3.2)	0.013
Diabetes	3,724 (28.6)	3,674 (28.2)	0.009
IBD	106 (0.8)	108 (0.8)	0.002
Renal disease	2,457 (18.8)	2,436 (18.7)	0.004
Laboratory test results			
Hemoglobin, g/dL	11.1 (9.5–12.9)	11.0 (9.5–12.8)	0.004
While blood cells, K/uL	13.0 (9.3–18.2)	13.3 (9.4–18.6)	0.006
Albumin, g/dL	3.1 (2.6–3.6)	3.1 (2.5–3.6)	0.007
Creatinine, mg/dL	1.3 (0.9–2.2)	1.3 (0.9–2.3)	0.001
ALT, U/L	27.0 (16.0–51.0)	28.0 (17.0–57.0)	0.005
AST, U/L	37.0 (22.0–81.0)	40.0 (24.0–91.0)	0.011
Total bilirubin, mg/dL	0.7 (0.4–1.3)	0.8 (0.5–1.5)	0.010
Treatments, n (%)			
Metronidazole	2067 (15.8)	2013 (15.4)	0.011
Vancomycin	4,724 (36.2)	4,639 (35.6)	0.013
High risk antibiotics	7,024 (53.8)	6,925 (53.1)	0.008
Immunosuppression	3,293 (25.2)	3,221 (24.7)	0.013

Abbreviations: PPI, proton pump inhibitors; SMD, standardized mean difference; BMI, body mass index; AIDS, acquired immunodeficiency syndrome; CHF, congestive heart failure; COPD, chronic obstructive pulmonary disease; IBD, inflammatory bowel disease; ALT, alanine aminotransferase; AST, aspartate aminotransferase.

### 3.2 Outcomes and subgroup analyses

**FIGURE 2:**
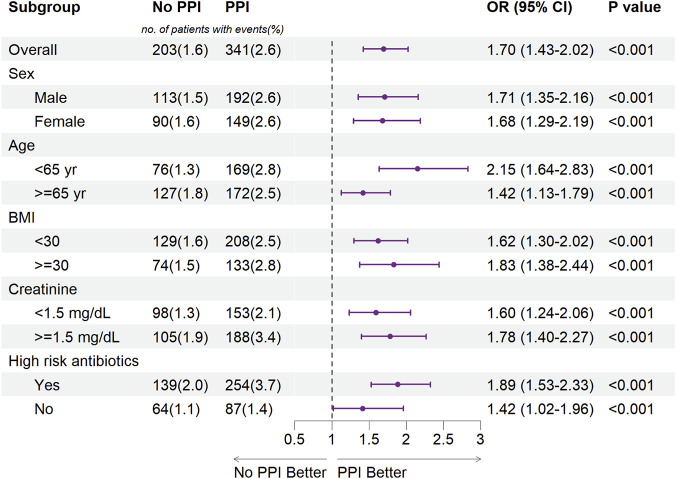
Risk of CDI between PPI-Naive and PPI-Exposed patients and subgroup analysis. Abbreviations: CDI, *Clostridioides difficile* infection; PPI, proton pump inhibitors; BMI, body mass index; OR, odds ratio; CI, confidence interval.

After propensity score matching, 341 patients in the PPI group developed CDI during ICU hospitalization, compared with 203 patients in the non-PPI group. The incidence of CDI was significantly higher in the PPI group than in the non-PPI group (PPI vs. No-PPI: 2.6% vs. 1.6%; OR, 1.70; 95% CI, 1.43–2.02; p < 0.001) (Figure 2). We conducted subgroup analyses by sex, age, BMI, creatinine and high risk antibiotics exposure. Across all subgroups (male, female, age < 65 years, age ≥ 65 years, BMI < 30, BMI ≥ 30, creatinine < 1.5 mg/dL, creatinine ≥ 1.5 mg/dL, high risk antibiotics exposure, no high risk antibiotics exposure), the subgroup analysis results were consistent with the primary analysis, showing a significantly higher risk of CDI in the PPI group compared to the non-PPI group (Figure 2).

### 3.3 Risk factors during ICU

We performed multivariate logistic regression on baseline demographic characteristics, patient comorbidities, and treatments received to explore independent risk factors for CDI. Obesity (BMI ≥ 30), cancer, diabetes, IBD, kidney disease, insulin exposure, and high-risk antibiotic exposure were significantly associated with increased CDI risk (Table 3). CHF increased CDI risk but was not statistically significant (OR, 1.19; 95% CI, 0.98–1.46; P, 0.084) (Table 3).

**TABLE 3:** Risk for *Clostridioides difficile* infection in the ICU.

Risk factors	CDI (%)	No CDI	OR (95%CI)	P
Age≥65 years	​	​	1.11 (0.97–1.27)	0.151
Yes	493 (1.6)	30,994	​	​
No	380 (1.4)	26,432	​	​
Sex	​	​	1.12 (0.98–1.28)	0.093
Male	460 (1.4)	31,910	​	​
Female	413 (1.6)	25,516	​	​
BMI≥30	​	​	1.40 (1.22–1.60)	<0.001
Yes	401 (1.8)	21,710	​	​
No	472 (1.3)	35,716	​	​
AIDS	​	​	1.76 (0.72–4.30)	0.206
Yes	5 (2.6)	187	​	​
No	868 (1.5)	57,239	​	​
Cancer	​	​	1.69 (1.39–2.06)	<0.001
Yes	116 (2.4)	4,764	​	​
No	757 (1.4)	52,662	​	​
CHF	​	​	1.13 (0.96–1.33)	0.138
Yes	189 (1.6)	11,278	​	​
No	684 (1.5)	46,148	​	​
COPD	​	​	1.18 (0.98–1.41)	0.074
Yes	148 (1.7)	8,491	​	​
No	725 (1.5)	48,935	​	​
Dementia	​	​	1.29 (0.87–1.91)	0.210
Yes	26 (1.9)	1,339	​	​
No	847 (1.5)	56,087	​	​
Diabetes	​	​	1.81 (1.56–2.11)	<0.001
Yes	242 (2.4)	10,021	​	​
No	631 (1.3)	47,405	​	​
IBD	​	​	2.76 (1.56–4.91)	0.001
Yes	13 (6.1)	201	​	​
No	531 (2.1)	25,345	​	​
Renal disease	​	​	2.18 (1.86–2.55)	<0.001
Yes	209 (2.8)	7,252	​	​
No	664 (1.3)	50,174	​	​
Insulin	​	​	1.36 (1.19–1.56)	<0.001
Yes	469 (1.7)	26,406	​	​
No	404 (1.3)	31,020	​	​
High risk antibiotics	​	​	2.11 (1.83–2.42)	<0.001
Yes	558 (2.1)	26,239	​	​
No	315 (1.0)	31,187	​	​
Immunosuppression	​	​	1.09 (0.93–1.28)	0.264
Yes	203 (1.6)	12,451	​	​
No	670 (1.5)	44,975	​	​

Abbreviations: CDI, *Clostridioides difficile* infection; OR, odds ratio; CI, confidence interval; BMI, body mass index; AIDS, acquired immunodeficiency syndrome; CHF, congestive heart failure; COPD, chronic obstructive pulmonary disease; IBD, inflammatory bowel disease.

## 4 Discussion

This study, based on two large, multi-center ICU databases in the United States, aimed to investigate whether PPI use increases the risk of CDI in critically ill patients. Our primary findings are as follows: (1) Among critically ill patients, PPI use significantly increased the risk of CDI compared with non-users; (2) Across all subgroups (male, female, age < 65 years, age ≥ 65 years, BMI < 30, BMI ≥ 30, creatinine < 1.5 mg/dL, creatinine ≥ 1.5 mg/dL, high risk antibiotics exposure, no high risk antibiotics exposure), PPI use was similarly associated with a significantly increased risk of CDI; (3) Obesity, cancer, diabetes, IBD, kidney disease, insulin exposure, and high-risk antibiotic use were independent risk factors for CDI.

Our findings indicate that PPI use significantly increases the risk of CDI in critically ill patients. An open-label crossover trial compared microbial community changes between subjects’ baseline period and during PPI treatment. They found that during PPI therapy, significant alterations occurred in both CDI-associated taxa (increased Enterobacteriaceae and Streptococcaceae, decreased Clostridiales) and taxa linked to gastrointestinal bacterial overgrowth (increased Micrococcaceae and Staphylococcaceae), alongside increased expression of genes involved in bacterial invasion (Freedberg et al., 2015). Research findings indicate that PPIs may increase the risk of CDI by altering key microbial populations that help resist *Clostridioides difficile* colonization. Other studies have also demonstrated that PPIs can cause gastrointestinal dysbiosis, thereby increasing susceptibility to CDI (Imhann et al., 2016; Zhang et al., 2023; Imhann et al., 2017).

Additionally, PPIs elevate gastric pH, which may also enhance the infectivity of *Clostridioides difficile*. Depending on the PPI type, the acid-reducing effect of a single daily dose can persist for 1–3 days, after which gastric pH gradually returns to baseline levels (Shin and Kim, 2013). A retrospective cohort study demonstrated a positive correlation between CDI risk and PPI dosage, with higher-dose PPIs associated with greater CDI risk than medium-dose PPIs. This suggests that elevated pH levels associated with high-dose therapy may increase CDI susceptibility (Park et al., 2019).

We also found obesity to be an independent risk factor for CDI. Results from multiple prior retrospective cohort studies align with ours, indicating that obesity increases the risk of CDI (Bishara et al., 2013; Atamna et al., 2025). This may be due to obesity’s impact on the gut microbiota, leading to reduced diversity and alterations in specific bacterial communities. A systematic review of 32 studies examined differences in gut microbiota between obese and normal-weight individuals, revealing a higher Firmicutes/Bacteroidetes ratio in obese patients (Crovesy et al., 2020). Research suggests that dysbiosis may predispose obese individuals to *Clostridioides difficile* colonization and infection (Spigaglia, 2024).

Furthermore, obese patients typically exhibit elevated levels of inflammatory cytokines and other immune components in their serum, placing the body in a state of chronic inflammation (Ellulu et al., 2017). A prospective cohort study demonstrated that this inflammatory state increases the risk of CDI (El Feghaly et al., 2013). Thus, obese patients are not only more susceptible to CDI due to altered gut microbiota but also face heightened infection risk from the inflammatory environment associated with obesity.

Studies indicate that the incidence of CDI and readmission rates among diabetic patients are significantly higher than in non-diabetic patients, consistent with our findings (Sharma et al., 2021; López-de-Andrés et al., 2018). The markedly increased susceptibility of diabetic patients to CDI results from the combined effects of multiple factors. Prolonged exposure to hyperglycemia in diabetic patients leads to persistent immune dysfunction, weakening the body’s ability to defend against pathogens, particularly those causing intestinal infections. Hyperglycemia compromises immune function by impairing neutrophil and macrophage activity, weakening the mucosal barrier, and promoting chronic inflammation, thereby increasing susceptibility to bacterial infections, including CDI (Darwitz et al., 2024). We found that insulin exposure is associated with an increased risk of CDI. However, this association may be due to the patients’ diabetes rather than the direct pharmacological effects of insulin.

Our findings indicate that inflammatory bowel disease (IBD) and kidney disease also increase the risk of *Clostridioides difficile* infection (CDI). The risk of CDI in IBD patients is many times higher than in the general population (Khanna, 2021). This susceptibility stems from frequent hospitalizations, antibiotic use, disruption of the gut microbiota, compromised mucosal barrier integrity, and impaired immune function (Ananthakrishnan, 2011; Issa et al., 2007). Active IBD manifests as chronic mucosal inflammation, increasing intestinal permeability, and reducing protective mucus and antimicrobial peptide production. These alterations allow *Clostridioides difficile* toxins to directly contact epithelial cells, thereby exacerbating inflammation and tissue damage (Hu et al., 2023). The higher incidence of ICD in patients with kidney disease may be associated with their impaired immune function (Thongprayoon et al., 2015; Goyal et al., 2017).

This study has several strengths and limitations. Data were extracted from the eICU and MIMIC IV databases, which draw from multi-center, large-sample populations, lending our data and findings a degree of representativeness. This constitutes a key strength of our study. A limitation of this research is its retrospective nature, which inevitably introduces certain biases. Furthermore, while propensity score matching effectively balances confounding factors among measured variables, it cannot address biases arising from unmeasured variables. Thirdly, due to the unavailability of pre-admission medication data, a small proportion of patients may have been misclassified. Finally, our analysis did not model PPI exposure as a time-dependent variable, which may have introduced residual immortal time bias.

Among critically ill patients, PPI recipients exhibited a significantly increased risk of CDI compared to those not using PPIs.

## Data Availability

Publicly available datasets were analyzed in this study. This data can be found here: eICU: https://physionet.org/content/eicu-crd/2.0/; MIMIC IV: https://physionet.org/content/mimiciv/3.1/; accession number: 11678655.
