# Supplementary material for: Proton pump inhibitors and the risk of hospital acquired clostridioides difficile infection in critically ill patients
Source: Front Pharmacol. 2026 Jul 13;17:1829764. doi: 10.3389/fphar.2026.1829764 (PMC13402174; doi:10.3389/fphar.2026.1829764)
Supplement: Supplementary file 1 [file Image1.pdf]

Supplemental Content

Figure S1: Percentage of missing variables.

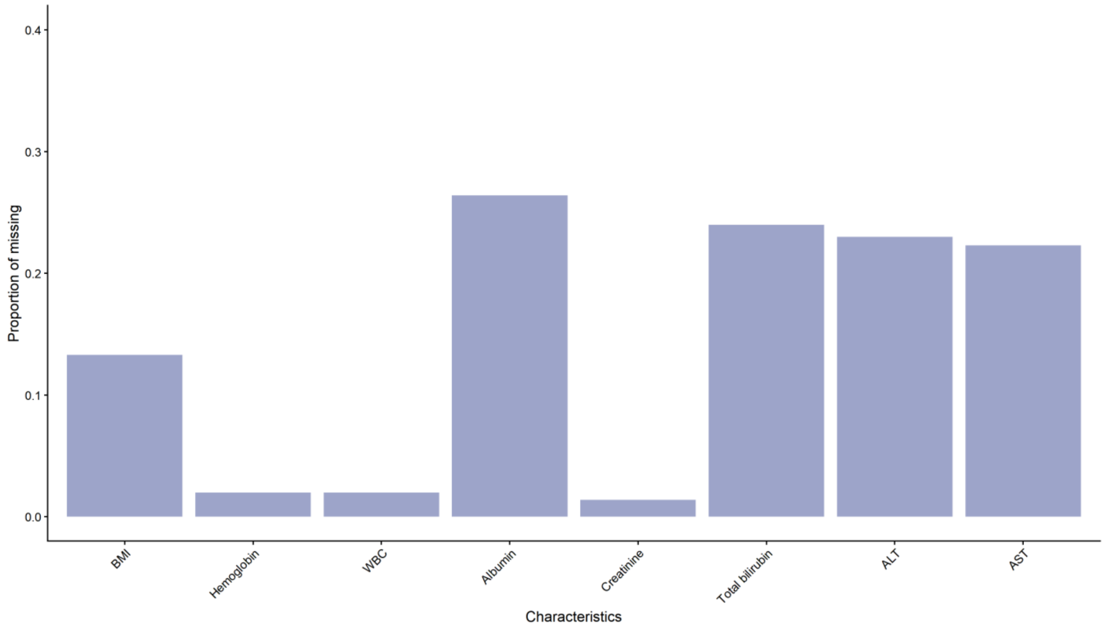

In Figure S1, we summarized the percentage of missing data. The missing percentages for each variable were as follows: BMI (13.3%), Hemoglobin (2.0%), WBC (2.0%), Albumin (26.4%), Creatinine (1.4%), Total bilirubin (24.0%), ALT (23.0%), AST (22.3%). Abbreviations: BMI, body mass index; WBC, white blood cell; ALT, alanine aminotransferase; AST, aspartate transaminase.
